# Crystal Structure of PrgI-SipD: Insight into a Secretion Competent State of the Type Three Secretion System Needle Tip and its Interaction with Host Ligands

**DOI:** 10.1371/journal.ppat.1002163

**Published:** 2011-08-04

**Authors:** Michele Lunelli, Robert Hurwitz, Jutta Lambers, Michael Kolbe

**Affiliations:** 1 Max Planck Institute for Infection Biology, Department of Cellular Microbiology, Berlin, Germany; 2 Max Planck Institute for Infection Biology, Core Facility Protein Purification, Berlin, Germany; University of California San Diego, United States of America

## Abstract

Many infectious Gram-negative bacteria, including *Salmonella typhimurium,* require a Type Three Secretion System (T3SS) to translocate virulence factors into host cells. The T3SS consists of a membrane protein complex and an extracellular needle together that form a continuous channel. Regulated secretion of virulence factors requires the presence of SipD at the T3SS needle tip in *S. typhimurium*. Here we report three-dimensional structures of individual SipD, SipD in fusion with the needle subunit PrgI, and of SipD:PrgI in complex with the bile salt, deoxycholate. Assembly of the complex involves major conformational changes in both SipD and PrgI. This rearrangement is mediated via a π bulge in the central SipD helix and is stabilized by conserved amino acids that may allow for specificity in the assembly and composition of the tip proteins. Five copies each of the needle subunit PrgI and SipD form the T3SS needle tip complex. Using surface plasmon resonance spectroscopy and crystal structure analysis we found that the T3SS needle tip complex binds deoxycholate with micromolar affinity via a cleft formed at the SipD:PrgI interface. In the structure-based three-dimensional model of the T3SS needle tip, the bound deoxycholate faces the host membrane. Recently, binding of SipD with bile salts present in the gut was shown to impede bacterial infection. Binding of bile salts to the SipD:PrgI interface in this particular arrangement may thus inhibit the T3SS function. The structures presented in this study provide insight into the open state of the T3SS needle tip. Our findings present the atomic details of the T3SS arrangement occurring at the pathogen-host interface.

## Author Summary

Since the rise of pathogenic bacterial strains resistant to antibiotics, the need to develop potent anti-infective drugs is continually increasing. This necessitates a detailed knowledge of the bacterial host invasion process. Gram-negative bacteria have evolved a protein transport system through which they deliver virulence factors into host cells. These virulence factors influence the signal transduction cascade and metabolism inside host cells in a way that is beneficial for the invading bacteria. The proteins at the transport system needle tip mediate contact with host cells and spatiotemporal coordinated release of virulence factors. In this study, we used biophysical and biochemical methods to understand the structure and function of proteins present at the needle tip of such a virulence factor transport system in *Salmonella* species. We could show that two different proteins, structurally conserved in many pathogenic bacteria, bind each other to constitute the needle tip of the transport system. Multiple copies of both proteins constitute the tip of the transport system in what may represent the open state of the needle. Our study will serve to provide new insights into the virulence factor transport system essential for many different pathogenic bacteria, and may thus offer novel targets to fight infection.

## Introduction

Bacterial infections including Salmonellosis and Shigellosis affect millions of people every year. These bacteria use a T3SS to secrete virulence factors to manipulate host cells. The T3SS is a multi-component system that forms a continuous protein transport channel through the two bacterial membranes and the periplasmatic space that extends into the surrounding medium by a needle structure [1]–[3]. Spatiotemporal control of secretion is essential for effective host invasion [4].

Tip proteins, which bind to the distal end of the T3SS needle, are thought to play an important role in this process [4]–[6]. SipD from *S. typhimurium*, IpaD from *Shigella flexneri* and BipD from *Burkholderia mallei* are tip proteins that are thought to interact with their corresponding needle subunits PrgI, MxiH and BsaL, respectively, to make the needle tip complex [5]. Although the mechanism is unclear, tip proteins were shown to influence secretion and invasion of bacteria [7]–[9].

Sterols like cholesterol or cholic acid derivatives found in the bile are amphipathic compounds that play important roles in cellular communication and metabolic processes. Bile salts influence the T3SS of intestinal bacteria. For instance, the presence of deoxycholate either impedes (*S. typhimurium*) or facilitates (*S. flexneri*) host invasion[10]–[13]. Noteworthy, it was recently shown that SipD and IpaD bind deoxycholate and some of its derivatives [14], [15].

To understand how T3SS are regulated it is required to analyze the structure and mechanism of proteins gating the transport channel. Here, we address the questions of how the Salmonella SipD interacts with PrgI and deoxycholate and the mechanistic consequences of the assembly of the T3SS needle tip complex.

## Results

### Crystal structure of the Salmonella tip protein SipD

**Figure 1:**
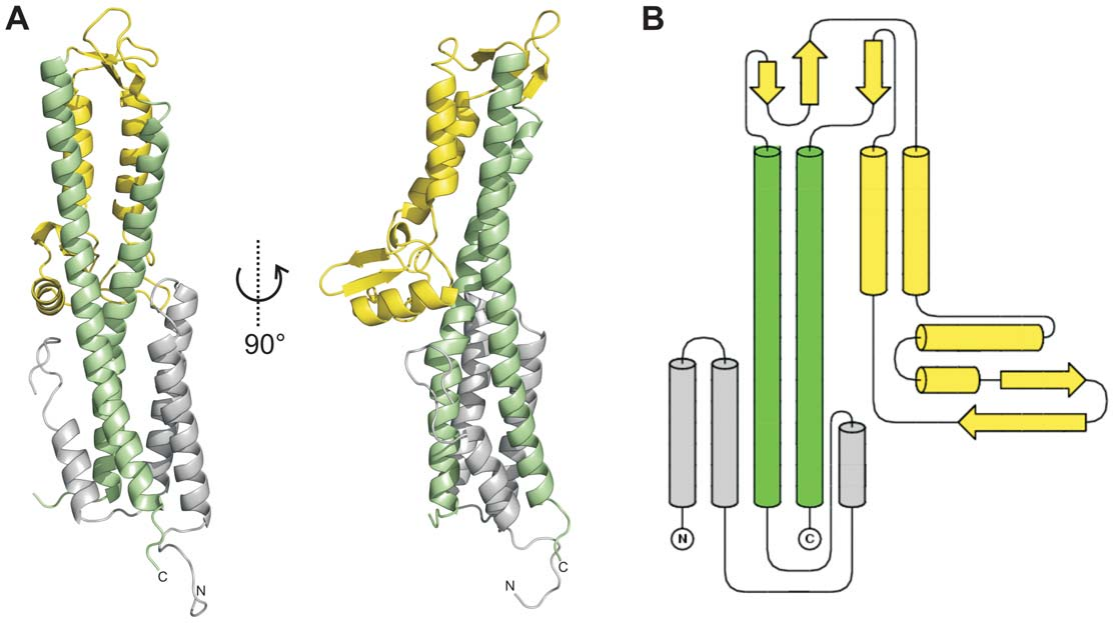
Crystal structure of SipD. (A) X-ray crystal structure and (B) topology plot of SipD chain A from *S. typhimurium*: structurally distinguishable domains are coloured in grey (domain 1), green (domain 2), and yellow (domain 3), respectively.

We solved the X-ray crystal structure of SipD, the needle tip protein of *S. typhimurium* at 3.0 Å resolution (Figure 1A and Table 1). The crystal contained 4 copies of SipD in the asymmetric unit with structural information for 306 of 343 amino acids. In SipD crystals, as in the crystal structures of IpaD [16] and BipD [17], the N-terminal 31, 38 or 29 amino acids, respectively, were not defined. Here we report structural features of SipD chain A, which, compared with chains B to D, showed continuous electron density for most amino acids. SipD is predominantly α-helical folded and can be divided in three structurally different domains (Figure 1B). The central domain, domain 2, (green in Figure 1) adopted a coiled coil structure with two helices of 47 and 52 amino acids length, respectively. The bending of the coiled coil of domain 2 allowed us to distinguish between a concave and a convex surface. Domain 2 is joined to domain 3, an α/β-structure (yellow in Figure 1). Domain 3 is in contact with the convex surface provided by the coiled coil of the central domain (Figure 1A). Domain 2 and 3 of SipD share high sequence homology and similar three dimensional structure to the orthologs from *S. flexneri* (Figure S1, r.m.s. deviation 1.3 Å with IpaD) [16] or *B. mallei* (Figure S1, r.m.s. deviation 2.2 Å with BipD) [17]. Notably, amino acids involved in intramolecular contacts are identical or highly conserved in all three orthologs, suggesting functional relevance in T3SS tip proteins.

**Table 1:** Data collection and refinement statistics.

	SipD_ΔD1_	SipD	PrgI-SipD_ΔD1_	PrgI-SipD_ΔD1_ + DXC
**Data collection**				
Space group	P2_1_2_1_2_1_	P6_5_22	C2	C2
Cell dimensions				
*a*, *b*, *c* (Å)	55.8, 94.4, 117.7	128.4, 128.4, 350.1	172.2, 48.1, 103.7	171.2, 47.5, 102.6
α, β, γ (°)	90, 90, 90	90, 90, 120	90, 121.9, 90	90, 122.0, 90
Resolution (Å)	40.00–3.00 (3.15–3.00)	20.00–3.00 (3.17–3.00)	40.00–2.40 (2.54–2.40)	40.00–2.19 (2.26–2.19)
*R_sym_*	0.063 (0.473)	0.098 (0.508)	0.081 (0.498)	0.070 (0.481)
*I/σ(I)*	19.77 (3.42)	13.86 (3.58)	12.47 (2.97)	16.02 (4.73)
Completeness (%)	99.3 (96.4)	96.7 (92.9)	97.4 (96.5)	95.4 (84.4)
Redundancy	5.0 (5.0)	7.6 (7.3)	3.7 (3.8)	5.8 (5.2)
**Refinement**				
Resolution (Å)	37.21- 3.00	19.94–3.00	32.89–2.40	36.48–2.19
No. reflections	12938	34082	27970	34906
*R*_work_/*R*_free_	0.198/0.224	0.229/0.258	0.222/0.251	0.246/0.284
No. atoms				
Protein	3065	7094	4116	4006
Ligand/ion	6	27	18	40
Water	21	38	67	97
*B*-factors				
Protein	64.5	80.9	47.9	53.4
Ligand/ion	77.9	89.3	46.4	57.9
Water	47.6	59.4	38.9	45.0
R.m.s. deviations				
Bond lengths (Å)	0.007	0.008	0.006	0.007
Bond angles (°)	1.1	1.4	1.1	1.1

Values in parentheses are for highest-resolution shell.

Abbreviations:

SipD_ΔD1_: N-terminal truncated SipD (SipD_132–343_).

PrgI-SipD_ΔD1_: fusion protein PrgI + truncated SipD (see Methods).

DXC: deoxycholate.

In contrast to domain 2 and 3, the amino acid sequence of domain 1 in SipD (grey in Figure 1) show almost no sequence conservation with IpaD or BipD. However, α-helices of domain 1 adopted a similar structure in both SipD and IpaD (Figure S2), encompassing the central coiled coil of domain 2 (Figure 1A).

### PrgI displaces the N-terminal domain of SipD

**Figure 2:**
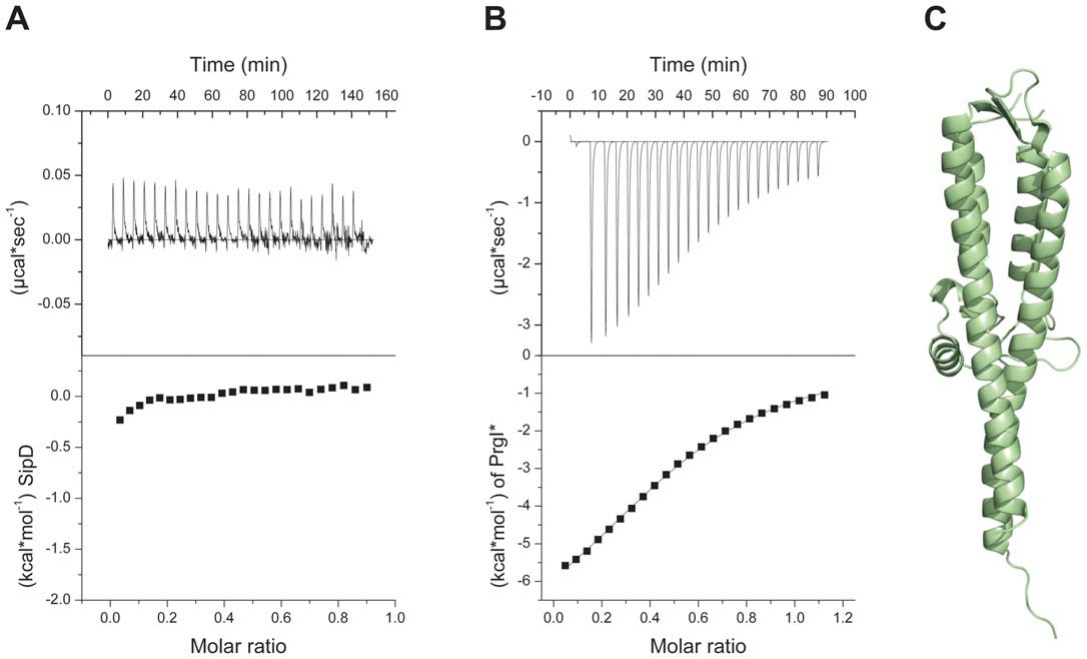
Deletion of SipD domain 1 permits binding to PrgI*. (A) Binding between *S. typhimurium* needle protein PrgI* and SipD was not detectable using isothermal titration calorimetry (ITC). Heat flow for each SipD injection as a function of time (upper) and integration of the thermogram (lower). (B) ITC of purified proteins PrgI* with SipD_ΔD1_ indicated a binding affinity of 88±3 µMol. Heat flow for each PrgI* injection as a function of time (upper) and integration of the thermogram with the fitting curve (lower). (C) X-ray crystal structure of SipD_ΔD1_.

In the *S. flexneri* cytosol, IpaD domain 1 was suggested to function as a self-chaperone avoiding either spontaneous self oligomerization or its interaction with the needle forming protein MxiH before secretion [16]. We tested the oligomerization state of purified SipD by static light scattering and found that SipD is a monomer in solution (M_w_ ∼37 kDa, Figure S3). Deletion of domain 1 (SipD_ΔD1_) resulted in a mixture of SipD dimers and trimers (Figure S4). Though oligomerization of the needle tip protein changed depending on the presence of domain 1, deletion of this domain did not favour spontaneous protein polymerization as was found for PrgI and its orthologues [18]. SipD did not bind to PrgI*, a soluble and functional PrgI mutant [18], as tested by isothermic titration calorimetry (ITC, Figure 2A). In contrast, SipD_ΔD1_ bound with a K_d_ of 88±3 µM to PrgI* (Figure 2B). Deletion of domain 1 did not destabilize SipD, as demonstrated by the comparative analysis of X-ray crystal structures of SipD_ΔD1_ (Figure 2C and Table 1) and SipD (Figure 1A). Superposition of SipD_ΔD1_ and SipD showed almost identical 3-dimensional structure reflected in an r.m.s. deviation of 0.9 Å for amino acids 149 to 328. ITC results showed that domains 2 and 3 of SipD mediate binding to PrgI*, while domain 1 impedes the interaction. In fact, prior to binding to PrgI*, the self-chaperoning domain 1 of SipD may unfold independently from the rest of the molecule as recently suggested [19], [20].

### Structure of the PrgI-SipD_ΔD1_ fusion protein

**Figure 3:**
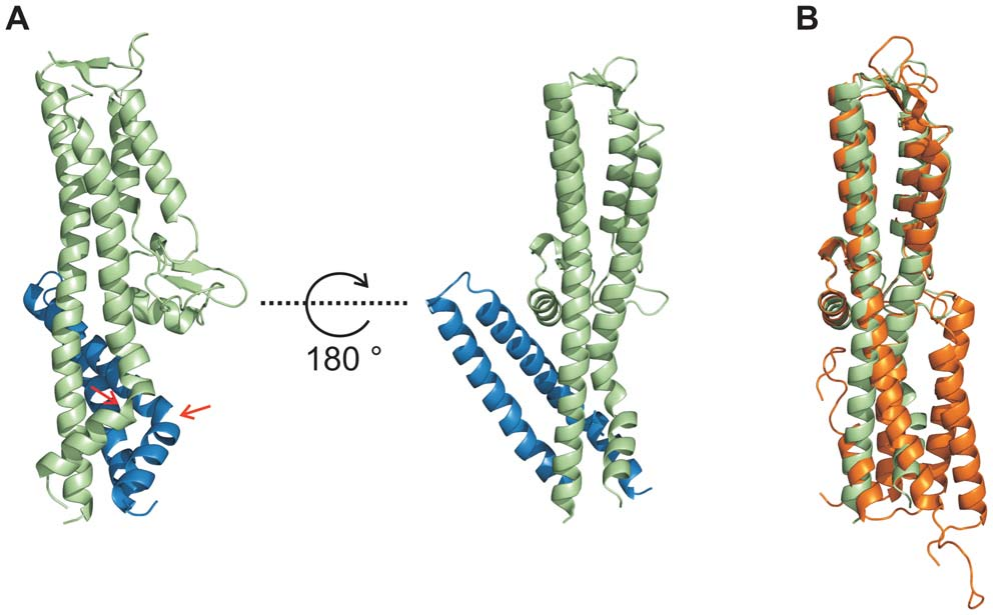
Conformational changes during interaction of SipD and PrgI. (A) Structure of the PrgI-SipD_ΔD1_ fusion protein (SipD green and PrgI blue) from two orthogonal perspectives. The helix kink in SipD and PrgI is indicated by red arrows. (B) Superposition of complexed SipD (green) with monomeric SipD (orange) indicated conformational changes in the central coiled coil.

In order to decipher the 3-dimensional structure of the entire T3SS needle tip, we generated a fusion protein of N-terminal truncated SipD with PrgI (PrgI-SipD_ΔD1_). Crystals of PrgI-SipD_ΔD1_ contained two similar copies (chain A and chain B) in the asymmetric unit. We describe here the structure of chain A, which is more complete than chain B. The X-ray crystal structure of PrgI-SipD_ΔD1_ solved at 2.4 Å resolution showed noteworthy features (Figure 3A and Table 1). Comparing structures of individual SipD (brown) and PrgI* molecules (grey) with the PrgI-SipD_ΔD1_ fusion protein (SipD_ΔD1_ in green and PrgI in blue) revealed conformational changes in both proteins (Figure 3B and Figure S5). Two of the five helices providing contact between SipD and PrgI changed conformation during complex formation. The central helix of domain 2 in SipD is kinked at Asn141 in the complex (Figure 3A–B). We observed also a kink in the C-terminal helix of PrgI at Asn63 (Figure 3A and Figure S5). Both kinked helices, together with two additional helices from SipD and PrgI, adopted a new four helix bundle in the complex.

Recent studies [9] suggest that the needle proteins could replace the two helices of domain 1 in the corresponding T3SS tip protein that are in contact with the concave side of the central coiled coil (Figure 1A). This hypothesis is in agreement with our ITC data showing that PrgI* may replace domain 1 during SipD binding (Figure 2A–B). However, the crystal structure of the complex presented here revealed PrgI binding to the convex surface of the central coiled coil in SipD (Figure 3A). Thus, in contrast to the proposed model, a single PrgI molecule may replace the helix-loop motif of SipD immediately upstream of domain 2 (Figure 1B), instead of the two helices at the N-terminus of SipD. Moreover, both proteins in the complex comprised an angle of about 45° (Figure 3A and Figure S6) due to the contact between SipD domain 3 and PrgI.

We tested the oligomerization state of the PrgI-SipD_ΔD1_ fusion protein using static light scattering technique. The needle tip complex was monomeric in solution (Figure S7), suggesting that the supramolecular architecture of the tip complex is influenced by the PrgI assembly of the T3SS needle. This hypothesis is in agreement with our observation that deletion of domain 1 of SipD did not support polymerization of the needle tip protein (Figure S4) but rather allowed interaction with the needle protein PrgI (Figure 2).

### Stabilization of the PrgI-SipD_ΔD1_ fusion protein

**Figure 4:**
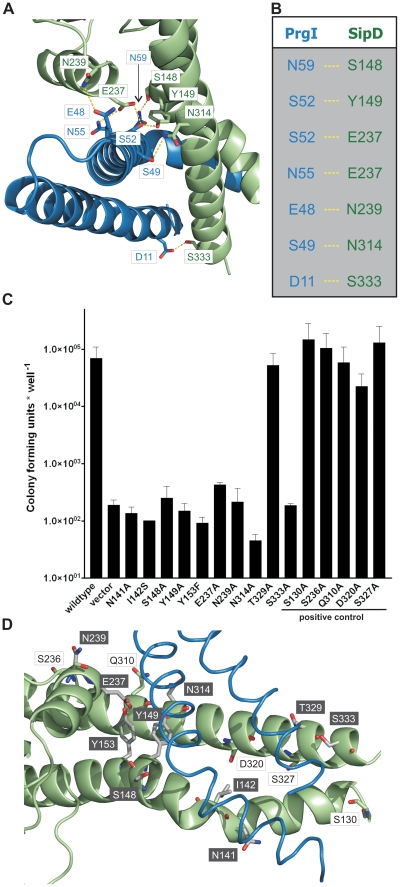
Interaction of SipD and PrgI in the T3SS needle tip complex. (A) Four helix bundle of the tip complex formed by two helices of SipD domain 2 (green) and the helix termini of PrgI (blue). Side chains that provided hydrogen bonding network (yellow) between PrgI-SipD_ΔD1_ in the fusion protein are highlighted. (B) List of the amino acids that stabilized the PrgI-SipD_ΔD1_ fusion protein by forming hydrogen bonds. (C) Host invasion assay of SipD mutant complemented *S. typhimurium* knockout cells. (D) Close up of the PrgI-SipD_ΔD1_ interface: mutated SipD amino acids interacting with PrgI are highlighted in grey as stick model and corresponding labels marked with grey boxes.

The X-ray crystal structure of the PrgI-SipD_ΔD1_ complex compared with the structure of SipD alone and with previous structural studies of needle tip proteins [18], [21]–[23] showed that both PrgI and SipD changed conformation during complex assembly. As mentioned above, the two helices which showed novel kinked conformation are part of a four helix bundle, thus providing close contact between SipD and PrgI (Figure 3A and Figure 4A). The four helix bundle stabilized by hydrophobic (Figure S8) and polar interactions encompassed a buried surface of 1113 Å^2^ per protein. An extended hydrogen bonding network connecting conserved amino acids of both proteins stabilizes the twisted helical arrangement found in the PrgI-SipD_ΔD1_ fusion protein (Figure 4A). In total six hydrogen bonds and salt bridges between the C-terminal helix of PrgI and the long helices of domain 2 or the central helix of SipD domain 3 stabilized the tertiary structure of the complex (Figure 4B). A hydrogen bond between Ser333 and Asp11 of SipD and PrgI, respectively, contributed additional stabilization of the complex structure. Spin labelled amino acids Asp136, Ala144, Asp147, Leu318, Lys324, Ser328, Ser331 and Glu335 of SipD are influenced by PrgI as recently shown [24], consistently with the PrgI-SipD_ΔD1_ crystal structure presented here.

We tested whether the interactions between SipD and PrgI are necessary for the T3SS function in HeLa cell invasion assays. SipD knockout cells, which are not invasive, were complemented with plasmids harbouring wildtype or mutant *sipD* (Figure 4C). We designed *sipD* mutants based on the structure of the PrgI-SipD_ΔD1_ fusion protein and on conservation of SipD amino acids (Figure S1). Including control mutants that did not hamper stabilization of the tip complex, we tested 15 SipD point mutations (Figure 4C). Except for Tyr153 and Glu237, which formed intramolecular hydrogen bonds, tested SipD mutants should not destabilize folding of the apo-protein. Most interactions between SipD and PrgI were essential for a functional T3SS as demonstrated by a dramatically reduced invasiveness (Figure 4C). In contrast, amino acids in the same region of SipD not interacting with PrgI had little or no effect (S130A, S236A, Q310A, D320A, S327A) on host cell invasion (Figure 4C–D). Circular dichroism spectroscopy of purified mutants and wildtype SipD indicated same folding, albeit the weaker spectrum of I142S indicates less α-helical content and suggests lower stability at 37°C (Figure S9). Similarly *prgI* mutant complemented PrgI knockout cells showed reduced invasiveness (Figure S10). Our results showed that mutations in the C-terminus of needle proteins can impede host invasion and are in agreement with previous studies in *S. typhimurium* and other T3SS dependent bacteria [21]–[23], [25].

### π–bulge in SipD provides conformational flexibility

We showed that polar interactions at the PrgI-SipD_ΔD1_ interface provide tight and specific contacts between the needle tip complex components. In close proximity of these contacts two helices adopted kinked conformation in the complex, but not in the individual proteins (Figure 3). By comparing the structures of PrgI-SipD_ΔD1_ fusion protein and SipD, we can propose how assembly of the T3SS needle tip is established.

**Figure 5:**
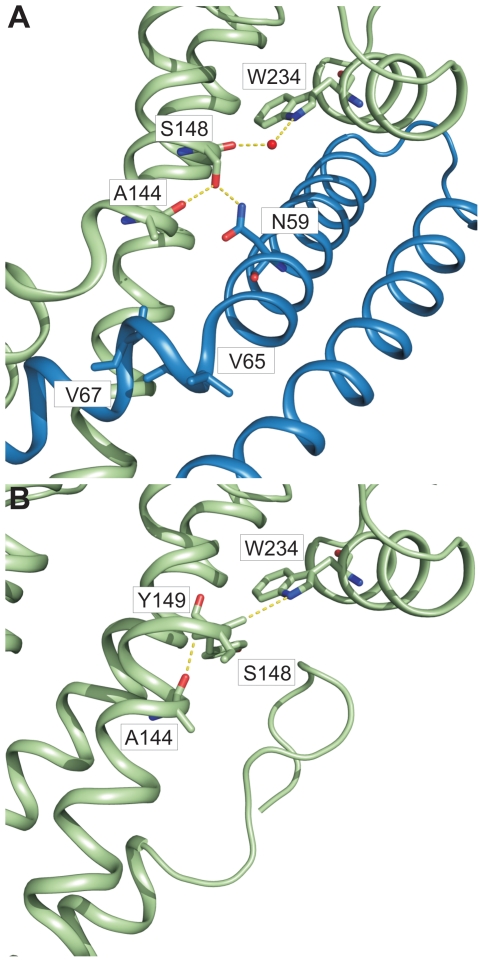
π–bulge in SipD domain 2 permitted the assembly of the PrgI-SipD_ΔD1_ complex. Amino acids (stick model) and interactions (yellow) that stabilize the π–bulge and helix kinks in the PrgI-SipD_ΔD1_ fusion protein (A) and SipD (B) are highlighted.

In chain A of both structures, one helix of the coiled coil (domain 2 of SipD) showed partial unwinding. This helix anomaly, usually energetically disfavoured, is stabilized in the fusion protein by hydrogen bonds formed between carbonyl oxygen of amino acid Ser148, a water molecule, and Trp234 of SipD (Figure 5A). The interaction of the Ser148 backbone carbonyl group with Trp234 shifted the typical backbone hydrogen bonding pattern stabilizing architecture of an α–helix (i→i+4) by one position (i(Ala144)→i+5(Tyr149)) (Figure 5B). Local deviation from the backbone hydrogen bonding pattern of an α–helix described as π-bulge plays an important role in many different proteins that require conformational flexibility for function [26]. In our structure of the PrgI-SipD_ΔD1_ fusion protein the π-bulge caused local unwinding and weakening of the α–helix around Ser148. Furthermore, mutation of either of the two amino acids (Ser148 or Trp234) involved in formation of the π-bulge led to reduced or even loss of bacterial invasion in HeLa cells (Figure 4C). These results support the relevance of the π-bulge for the functionality of the T3SS needle tip complex.

In the PrgI-SipD_ΔD1_ fusion protein a water molecule bridges the backbone carbonyl oxygen of Ser148 and Trp234 (Figure 5A). The backbone carbonyl of Ser148 pointing towards the imino group of Trp234 suggested similar hydrogen bond stabilization in SipD as in the PrgI-SipD_ΔD1_ fusion protein (Figure 5B). It is interesting to speculate whether the coiled coil of SipD is intrinsically destabilized even in the absence of PrgI based on the superposition of different SipD copies found in the SipD structure (Figure S11).

Structural flexibility introduced by the π-bulge may account partially for the helix kink observed in the complex. The SipD helix kink is stabilized through interactions between Ser148 side chain with Asn59 of PrgI, which is highly conserved in needle proteins (Figure 4A and Figure 5A). Notably, in the fusion protein the Ser148 side chain formed a hydrogen bond with the backbone carbonyl oxygen of Ala144, located one helix turn upstream (Figure 5A). This hydrogen bonding network stabilized the helix kink present at amino acid Ala144 of SipD in the complex. Taken together, a π–bulge in SipD may provide conformational flexibility to allow the formation of the T3SS needle tip complex. Conversely, conformational flexibility of SipD may be arrested by the folded domain 1 in cytosolic SipD.

As mentioned above, one helix of PrgI had a similar kink to SipD in the complex. Amino acids Val65 and Val67 of PrgI were located at the kink (Figure 5A). Interestingly, both amino acids were recently found to be critically involved in polymerization of the T3SS needle [18]. Briefly, substitution of valines at position 65 and 67 by alanines reduced polymerization kinetics of the needle protein, but did not abolish needle formation or affect bacterial host invasion. Assembly of the T3SS needle is coupled with α-helix-to-β-sheet change downstream of Val67. Noteworthy, both amino acids were located at the PrgI-SipD_ΔD1_ interface, suggesting a functional importance during assembly of the T3SS needle and tip complex. The visible amino acids downstream of the helix kink in PrgI adopted a helix conformation in the complex. Conformational flexibility, depending whether PrgI molecules interact with each other to form a needle or with SipD at the T3SS needle tip, may be a prerequisite for the function of this protein.

### PrgI-SipD_ΔD1_ fusion protein binds to deoxycholate

Host invasion of enteric bacteria is often dramatically influenced by the presence of bile salts [10]–[13]. Human intestinal bile salts, including deoxycholates, taurodeoxycholates and chenodeoxycholates, can either increase or repress invasion of *S. flexneri* or *S. typhimurium*, respectively [10], [14], [15]. The bile salt effect on those bacteria is coupled to a functional T3SS. Moreover, recent studies show that bile salts bind to needle tip proteins, corroborating that a T3SS component is affected by ligands released into the human gut [27].

**Figure 6:**
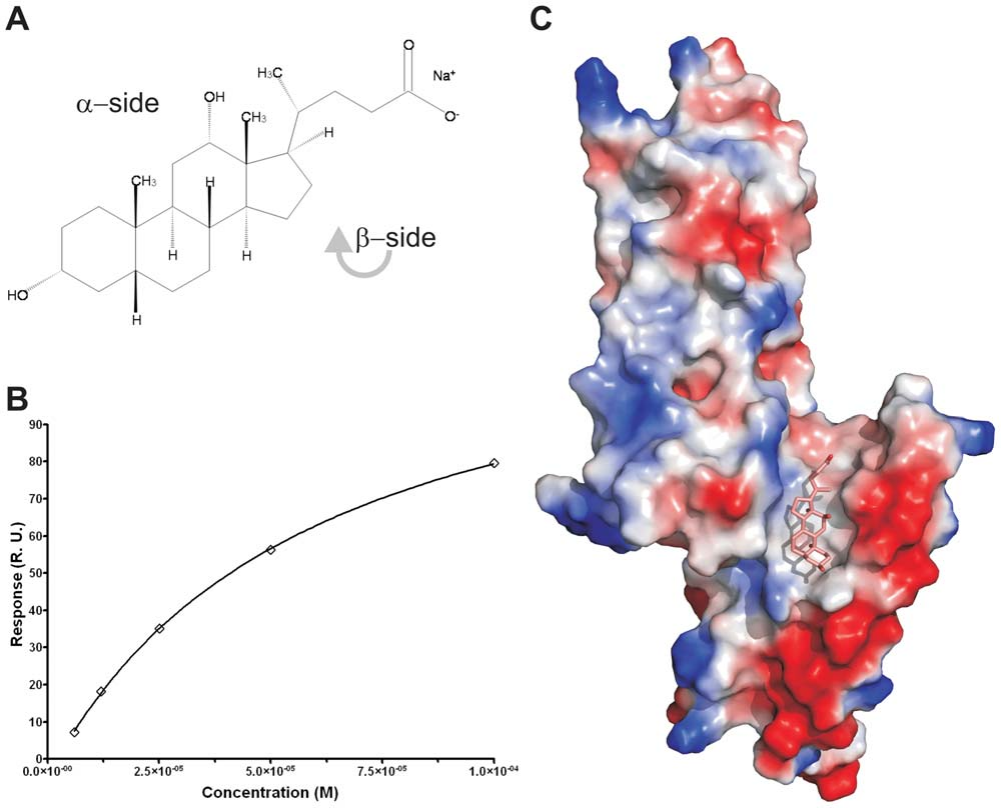
Binding assay and cocrystal structure of PrgI-SipD_ΔD1_ with deoxycholate. (A) Chemical formula of sodium deoxycholate, emphasizing the hydrophilic α- and the hydrophobic β-side of the molecule. (B) Binding curve of deoxycholate to PrgI-SipD_ΔD1_: plot of the equilibrium binding response versus the concentration of deoxycholate. The solid line represents the fitting curve of the individual measurements. (C) Surface representation of the PrgI-SipD_ΔD1_ fusion protein (chain B) coloured according the electrostatic potential (blue positive, red negative). Deoxycholate bound in the hydrophobic cleft of the PrgI-SipD_ΔD1_ complex through its β-side is highlighted as a stick model.

To measure whether the PrgI-SipD_ΔD1_ fusion protein could also bind deoxycholate (Figure 6A), we used surface plasmon resonance assay (Biacore). The binding curve of the immobilized PrgI-SipD_ΔD1_ fusion with increasing concentrations of deoxycholate (Figure 6B) indicated a dissociation constant of 59.0±2.7 µM, assuming a protein ligand ratio of 1 to 1. These data are in agreement with previous results indicating an affinity between the *S. flexneri* needle tip protein IpaD and deoxycholate in the micromolar range [14]. Next, we soaked PrgI-SipD_ΔD1_ crystals with sodium deoxycholate and analyzed the corresponding electron density for novel features that could fit the ligand. In the co-crystal structure (Figure 6C, Figure S12 and Table 1), deoxycholate was bound through its most hydrophobic β-surface (Figure 6A) to the cleft formed by SipD and PrgI. Localization of this binding site is about 25 Å away from the previously described deoxycholate binding site in wildtype SipD [28]. The α-surface of deoxycholate, disposing two hydroxyl groups, was facing the bulk medium. Ligand binding induced only minor structural changes related to the side chain of Ser236 in SipD and Gln24 and Gln48 in PrgI. Deoxycholate is a rigid molecule that fit almost perfectly into the cleft provided by the PrgI-SipD_ΔD1_ fusion protein. In the cocrystal, the deoxycholate carboxyl group located at the end of the binding cleft was not as tightly embedded as the rest of the ligand. Therefore bile salts with larger substituents at this position could also occupy the cleft. Indeed, taurodeoxycholate, which is deoxycholate amidated at the carboxyl group with ethansulfonic acid, also binds to SipD and IpaD [14], [15].

Results presented here are in agreement with recent NMR titration experiments showing that deoxycholate causes chemical shift changes in SipD upon binding to amino acids in the vicinity of the ligand binding site comprised by Arg232, Gln233, Ser236, Glu237 and Asn239 [15]. Moreover, mutation of amino acid Glu229 of IpaD, which is the equivalent of Glu237 in SipD, abolishes binding of deoxycholate [14].

Our data, however, indicate that bile salts bind to SipD similarly to the deoxycholate PrgI-SipD_ΔD1_ fusion protein. The inhibitory effect of this ligand protein interaction for host invasion suggests that other proteins need to bind to the hydrophobic cleft formed by SipD and PrgI.

## Discussion

We showed that the interaction of SipD with PrgI depends on the folding of domain 1 of SipD. In the PrgI-SipD_ΔD1_ fusion protein, PrgI replaces the helix of domain 1 of SipD forming a contact with the concave side of the coiled coil. Crystal structure analysis of both SipD and of PrgI-SipD_ΔD1_ reveal that the π-bulge in domain 2 of the tip protein contributes to the complex formation. Moreover, sterol binding to a cleft in the PrgI-SipD_ΔD1_ complex suggests that intestinal detergents released from the gallbladder could hamper regulated secretion of virulence factors and consequently prevent invasion of *S. typhimurium*.

The T3SS needle tip protein from *S. flexneri* and *Yersinia enterocolitica* shows a pentameric organization [29], [30]. We found that the PrgI-SipD_ΔD1_ fusion protein is monomeric in solution, suggesting that the architecture of the tip complex depends on the scaffold provided by the subunits of the T3SS needle.

### Three dimensional model of the T3SS needle tip

**Figure 7:**
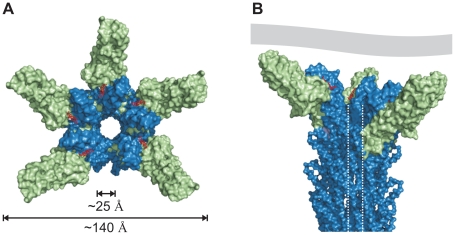
Model of the open state of the T3SS needle tip. (A) View from the surface into the channel opening of the T3SS needle tip. SipD is coloured in green and PrgI in blue, respectively. Deoxycholate molecules binding to PrgI-SipD_ΔD1_ are highlighted in red. The inner diameter of the needle tip complex (25 Å) permits passage of unfolded virulence proteins. Opening of the needle tip complex with an outer diameter of 140 Å results in a large inner cavity which could be closed through contact with the plasma membrane of host cells. (B) Side view of the T3SS needle including the tip complex. The inner channel is highlighted by dotted lines and a contacting host cell membrane is shown in grey.

The cryo-EM map of isolated needles from *S. flexneri* which is similar to the map obtained from isolated *S. typhimurium* needle [31] together with the X-ray crystal structure of a needle protomer mutant can be used to build a composite 3-dimensional model [21]. Based on this composite model of the T3SS needle we manually superimposed the similarly structured regions of the PrgI-SipD_ΔD1_ fusion protein with the MxiH subunits of the needle. Superposition of PrgI and MxiH (PDB code 2V6L) using the program Coot [32] was feasible without structural clashes. In total, five molecules of the PrgI-SipD_ΔD1_ fusion protein were successfully superimposed with five MxiH subunits at the distal end of the T3SS needle (Figure 7). As described above, we found that PrgI binds to the concave side of the central coiled coil in SipD. Therefore, the PrgI-SipD_ΔD1_ fusion protein could be mounted at the distal end of the needle without inducing structural changes. Domain1 present in SipD may face the bulk medium either in an unfold state or as a folded entity. In contrast to our model, the SipD-PrgI contact predicted by a previous work [9] would require substantial structural changes at the tip of the T3SS needle.

### Open state of the T3SS needle tip

The tip complex is the distal opening of the transport channel provided by the T3SS needle. According to the proposed model, SipD bound to PrgI localizes to the outer surface of the T3SS needle without obstructing the inner channel (Figure 7). The channel is opened in the three dimensional model of the T3SS tip complex (Figure 7A), adopting a state that permits transport or release of unfolded molecules after passage through the channel inside the needle. For this reason we assign the presented structure-based model as the “open state” of the T3SS needle tip. About 25 Å for the diameter of the T3SS channel may allow the passage of a single α-helix (or even of a helix-loop-helix motif).

Deletion of the T3SS needle tip protein causes constitutive secretion of virulence factors but abolishes bacterial invasion [7]. Consequently, it was proposed that the needle tip protein blocks the secretion of virulence factors. In contrast to this hypothesis, our structure based model of the open state suggests that the T3SS tip complex is not necessarily blocking the T3SS channel. Moreover, the tip protein does not need to be released for secretion of virulence factors, as the SipD-PrgI interaction is not clogging the channel. We speculate that the presence of the tip protein enables intermittent closing of the T3SS system thus regulating the process of secretion.

The three dimensional model presented here enables the following conclusions: The open state of the SipD-PrgI needle tip must be closed to block the constitutive transport of virulence factors. The closing of the T3SS needle tip can be mediated by either a conformational change of SipD or by its interaction with other effector proteins or lipids. Notably, the structure of the needle tip complex is not in conflict with possible movement of domain 2 and 3 in SipD but further work is required to explain how SipD regulates the secretion of T3SS. Moreover, binding of other effector proteins to the T3SS needle tip was proposed for the control of the needle length [1], [33] and the translocation of virulence factors across host membranes (SipB).

Along these lines, we previously reported that the T3SS needle protein PrgI extends the needle from the distal end in the absence of tip proteins [18]. Moreover, addition of tip proteins prevented further growth of the T3SS needle [18]. It is plausible that addition of SipD avoid needle elongation.

Bile salts, including deoxycholate, can prevent *S. typhimurium* invasion through binding to SipD [12], [15]. We showed here that deoxycholate binds to the cleft formed by SipD and PrgI close to the constriction of the T3SS channel (Figure 6 and Figure 7a). This interaction may prevent larger conformational changes in SipD, which block closure of the T3SS channel. Likewise bound deoxycholate may impede the binding of channel blocking proteins. Future studies are needed to understand how the T3SS is regulated using deoxycholate.

The presented structural studies enable us to construct a three dimensional model of the Salmonella T3SS needle tip, which in turn suggests a secretion mechanism. In the open state of the T3SS needle tip a large cavity, maybe enclosed through contact with the host membrane, is formed. This cavity could act as a folding chamber to facilitate the folding of secreted proteins. Folding of early secreted translocator proteins at the host membrane could improve the delivery of other effector proteins into the host cytoplasm. A similar folding principle was identified in the molecular chaperones, including prefoldin which forms a cavity for the nascent protein chain at the exit channel of the ribosome [34]–[36]. Moreover, the T3SS channel could be closed by contact with host membranes. In this scenario, the host membrane could prevent waste of secreted virulence factors, which otherwise could diffuse away from the point of contact. The T3SS needle tip is crucial for bacterial invasion and searching for substances similar to deoxycholate that prevent functioning or even assembly of the complex could lead to the discovery of novel targets for the development of drugs against pathogenic enterobacteria.

## Material and Methods

### Cloning, gene expression and protein purification

Wildtype and mutant *sipD* or *prgI* were amplified from *S. enterica* serovar *typhimurium* strain SL1344 (*S. typhimurium*) by standard PCR using oligonucleotide primers with *NdeI* and *XhoI* restriction sites at either ends. Single crystallization of SipD_132–343_ superseded cocrystallization with PrgI in various attempts. Therefore, wildtype *prgI* and a *sipD* fragment encoding amino acids 127 to 343 were connected by fusion PCR. N-terminal PrgI was fused by the amino acids Gly-Gly-Ser-Gly-Gly to SipD_127-343_.

PCR products were cloned into the expression vector pET-28a(+) (Novagen) or pET-21a(+) (Novagen), both containing N-terminal His-tag, and expressed in *Escherichia coli* BL21(DE3) cells. Cells were induced with isopropyl-β-D-1-thiogalactopyranoside, harvested after 4 h and His-tagged protein purified using affinity chromatography (HisTrap, GE Healthcare). Bound protein was washed (40 mM imidazole) and eluted using buffer containing 500 mM imidazole. After buffer exchange (20 mM HEPES pH 7.4, 50 mM NaCl) the tag was cleaved with CleanCleave Kit (Sigma-Aldrich). The cleaved product was purified by size-exclusion chromatography (Superdex 200 or Superdex 75, GE Healthcare) and stored at 4°C until use. For functional assays, wildtype or mutant *sipD* or *prgI* were cloned into the pASK-IBA5 vector (IBA) as *BsaI* fragments. Point mutants were generated using QuikChange Site-Directed Mutagenesis Kit (Stratagene). All constructs were confirmed by sequencing.

### Crystallization, data collection, structures determination and refinement

Crystals of SipD, SipD_ΔD1_ and PrgI-SipD_ΔD1_ were obtained at 18°C using hanging drop vapour diffusion technique. SipD was concentrated to ∼15 mg/ml and mixed with equal volume of reservoir solution containing 100 mM HEPES pH 7.5 and 1.5 M Li_2_SO_4_. SipD_ΔD1_ was concentrated to 40 mg/ml and mixed with equal volume of reservoir solution 0.1 M MES pH 6.5 and 12% (w/v) polyethylene glycol 20000. PrgI-SipD_ΔD1_ was concentrated to 30 mg/ml and mixed with equal volume of reservoir solution containing 0.49 M NaH_2_PO_4_ • H2O and 0.91 M K_2_HPO_4_, pH 6.9. To obtain cocrystals PrgI-SipD_ΔD1_ crystals were soaked for 72 hours in mother liquor containing ∼10 mM deoxycholate. All crystals were flash frozen in liquid nitrogen in the presence of 30% glycerol (v/v). Diffraction data were collected at 100 K and wavelength 0.918 Å at BESSY II (Berlin, Germany) beamlines 14.1 or 14.2, or wavelength 1.000 Å at SLS (Villigen, Switzerland) beamline X06SA.

Diffraction data were indexed, integrated and scaled using the program package XDS [37]. The crystal structure of SipD_ΔD1_ was solved by molecular replacement with the program Phaser [38] using the structure of truncated IpaD (pdb code: 2J0N) as template. The structures of SipD and PrgI-SipD_ΔD1_, apo and with deoxycholate, were solved by molecular replacement using the SipD_ΔD1_ structure as template. The initial models were refined by repeated cycles of manual building and refinement using the programs Coot [32] and CNS [39].

Crystals of SipD have 4 copies in the asymmetric unit and the following Ramachandran statistics: 82.7% of residues in most favoured regions, 16.6% in additionally allowed regions, 0.7% in generously allowed regions. Crystals of SipD_ΔD1_ have 2 copies in the asymmetric unit and 92.7% of residues in most favoured regions, 6.5% in additionally allowed regions, and 0.8% in generously allowed regions. Crystals of PrgI-SipD_ΔD1_ have 2 copies in the asymmetric unit and 92.9% of residues in most favoured regions, 7.1% in additionally allowed regions. The structure of PrgI-SipD_ΔD1_ complexed deoxycolate has 93.4% of residues in most favoured regions and 6.6% in additionally allowed regions. Ramachandran statistics were calculated with PROCHECK v.3.3 [40].

Molecular graphics images, including representations of surface electrostatic potential, were produced using PyMOL version 0.99rc6 [41], except Figure S6 which was produced with UCSF Chimera package from the Resource for Biocomputing, Visualization, and Informatics at the University of California, San Francisco [42].

### Generation of knockout strains

Bacterial knockouts were generated according to Datsenko and Wanner [43]. pASK-IBA5 plasmids harboring wild type or mutant *sipD* (p*sipD*) were used to complement deletions of *sipD* in *S. typhimurium* strain SL1344 to generate strains SL1344Δ*sipD/*p*sipD*.

### HeLa cell invasion assay

HeLa cells were seeded at 1×10^5^ cells per well and grown for 24 h at 37°C. Prior to infection, growth medium was aspirated, cells were washed twice with phosphate-buffered saline (PBS), and serum-free medium was added. To test for epithelial cell invasion and intracellular growth, HeLa cells were infected with *S. typhimurium* at a multiplicity of infection (MOI) of 10:1. Expression of *sipD wild-type* and *sipD mutants* was induced with 0.2 µg ml^−1^ anhydrotetracycline for 1 h. Bacterial inocula were prepared in PBS and centrifuged onto cells (2000 rpm, 10 min), and infected cultures incubated for 20 min at 37°C. Cultures were washed three times with PBS, and fresh medium containing 100 µg ml^−1^ gentamicin was added. After 2 h cells were washed with PBS and lysed with 0.1% Triton X-100. Numbers of viable bacteria were obtained by plating dilutions of lysates on tryptic soy agar plates and counting colonies after overnight incubation at 37°C.

### Multi-angle laser light scattering (MALLS)

For mass determination a combined setup consisting of SEC and subsequent online detection by UV absorption, (three angle) static laser light scattering and differential refractive index measurement was used as described earlier [44]. SEC was performed with either a Tricorn Superdex 200 10/300 GL column or a Tricorn Superdex 75 10/300 GL (GE Healthcare) equilibrated with 20 mM HEPES (pH 7.5), 150 mM NaCl. For static light scattering and differential refractive index measurements a linear coupled miniDAWN Tristar (Wyatt Technology) system and a differential refractive index detector (RI-101, Shodex), respectively, was used. All calculations were done with the software ASTRA (Wyatt Technology). Each experiment was repeated at least in triplicate.

### Isothermal Titration Calorimetry (ITC)

Titration experiments were carried out using a VP–ITC isothermal titration microcalorimeter (MicroCal, Northampton, MA, USA). Aliquots of 12 µl of SipD (1.35 mM) were injected consecutively at 20°C into the cell containing 1.4 ml of PrgI* (0.34 mM) or at 17°C by injecting consecutively 12 µl aliquots of PrgI* (1.99 mM) into the cell containing 1.4 ml of SipD_ΔD1_ (0.37 mM). The heat of dilution of the injected protein was measured in both cases and subtracted from the heath measured at each injection. Binding stoichiometry, enthalpy, and equilibrium association constants were determined by fitting the corrected data to one set of sites model equation using the evaluation software provided by the manufacturer.

### Surface plasmon resonance (Biacore)

Binding of sodium deoxycholate to the PrgI-SipD_ΔD1_ fusion protein was measured using surface plasmon technology-based Biacore X100 biosensor (GE Healthcare) according to manufacturer's instruction. Briefly, PrgI-SipD_ΔD1_ fusion protein was immobilized on a sensor chip CM5 (research grade) by amine coupling method. Binding experiments were performed at 25°C at continuous flow rate of HBS-N buffer (10 mM HEPES, 150 mM NaCl, pH 7.4). Deoxycholate was injected in steps with increasing concentrations in a single analysis cycle without regeneration of the surface in between injections. Affinity analysis was performed using single-cycle kinetics. Equilibrium dissociation constant (K_D_) was determined with Biacore evaluation Version 4.1 software. During the assays, the signal was corrected against the control surface response to eliminate refractive index changes due to buffer change.

### Circular dichroism spectroscopy

CD spectra were collected with a Jasco J-500A spectropolarimeter. Samples buffered in 10 mM HEPES (pH 7.4), 25 mM NaCl were measured either at 20°C and protein concentration1.5 mg/ml between 182 and 260 nm in a quartz cuvette with optical path length of 0.1 mm or at 37°C and protein concentration 0.15 mg/ml between 198 and 260 nm in a temperature controlled quartz cuvette with optical path length of 1 mm. Wavelength scans were carried out at a scan rate of 12 nm/min, with time constant 2 sec. All the spectra were acquired in triplicates.

### PDB accession codes

The atomic coordinates and structure factors of the four structures described here are available from the Protein Data Bank under the following accession codes: 2YM0 for SipD_ΔD1_, 2YM9 for SipD, 3ZQB for PrgI- SipD_ΔD1_, 3ZQE for PrgI- SipD_ΔD1_ complexed with deoxycholate.

## Supporting Information

Figure S1 **Structural conservation of T3SS needle tip proteins.** Superposition of the domains 2 and 3 of the crystal structures of SipD from *Salmonella* (orange, this work), BipD from *Burkholderia* (blue, PDB code 2IZP), and IpaD from *Shigella* (green, PDB code 2J0O) and corresponding structure based protein sequence alignment (below). Identical and similar amino acids are highlighted in dark and light grey, respectively. Amino acids are numbered according to the SipD sequence.
(TIF)

Figure S2 **Structural similarity of the tip protein in domain 1.** Superposition of the N-terminal domains of SipD (orange. this work) and IpaD (light blue, pdb code: 2J0O).
(PNG)

Figure S3 **SipD is a monomer in solution.** On-line static laser light scattering experiments of SipD eluted from a size exclusion column. The black line shows the protein absorption at 280nm (right axis) versus the eluted volume, indicating the presence of SipD. The light and dark grey lines refer to the left axis and reflect the measured molecular weight of SipD in solution for 2 independent experiments.
(TIF)

Figure S4 **Deletion of the N-terminal domain 1 in SipD does not facilitate self-polymerization.** On-line static laser light scattering experiments of SipD_ΔD1_ eluted from a size exclusion column. The black line show the protein absorption at 280nm (right axis) versus the eluted volume indicating the presence of SipD_ΔD1_. The light and dark grey lines refer to the left axis and reflect the measured molecular weight of SipD_ΔD1_ in solution for 2 independent experiments.
(TIF)

Figure S5 **Conformational changes during interaction of SipD and PrgI.** Superposition of PrgI as in the fusion protein (blue) with monomeric PrgI* (light brown) indicated structural differences in the C-terminal helix of the needle protein.
(TIF)

Figure S6 **Relative orientation of SipD and PrgI in the fusion protein.** SipD (green) and PrgI (blue) adopt a relative orientation of about 45°. Calculation is based on the relative orientation of the highlighted (grey cylinders) helices.
(PNG)

Figure S7 **PrgI-SipD_ΔD1_ fusion protein is a monomer in solution.** On-line static laser light scattering experiments of PrgI-SipD_ΔD1_ eluted from a size exclusion column. The black line show the protein absorption at 280nm (right axis) versus the eluted volume indicating the presence of PrgI-SipD_ΔD1_. The light and dark grey lines refer to the left axis and reflect the measured molecular weight of the fusion protein in solution for two independent experiments.
(TIF)

Figure S8 **Hydrophobic surfaces stabilize the PrgI-SipD_ΔD1_ fusion protein.** Two perspectives of SipD (green cartoon on the left, surface representation on the right) and PrgI (surface representation on the left, blue cartoon on the right). Surfaces are coloured according to the electrostatic potential, blue: positive, red: negative). Uncharged surface patches at the interface between the two proteins indicate hydrophobic contacts.
(TIF)

Figure S9 **Circular Dichroism spectra obtained at 20°C and 37°C from purified SipD and SipD mutants.** Except for I142S the spectra obtained from six mutants described in Figure 4 show similar secondary structure content at 20°C (upper panel) and at 37°C (lower panel). Mutant I142S shows reduced folding stability compared to wildtype, particularly at 37°C. Data at 37° (lower panel) were recorded to a lower limit of 1980 Å to avoid spectra distortion due to high photomultiplier voltage obtained with a temperature controlled cuvette with 1 mm optical path length.
(TIF)

Figure S10 **Host invasion assay of PrgI mutant complemented** ***S. typhimurium*** **knockout cells.**
(TIF)

Figure S11 **Superposition of the different copies found in the SipD crystal structure.** Chain A (orange), chain B (light blue), chain C (red), and chain D (green) were superimposed and the region of the coiled-coil around Ser148 is highlighted. Chains A and B show a π-bulge, chain C and D are partially destabilized and kinked at the same position as SipD in the fusion protein.
(PNG)

Figure S12 **Co-crystal structure of PrgI-SipD_ΔD1_ with deoxycholate.** Left: Ribbon presentation of the PrgI-SipD_ΔD1_ fusion protein (chain B, SipD_ΔD1_: green, PrgI: blue) in complex with deoxycholate (yellow); Right: Bound deoxycholate in the same orientation as on the left shown with superimposed composite 2fo-fc density map (blue) contoured at 1 σ.
(TIF)
