# Supplementary figures and images for: Crystal Structure of PrgI-SipD: Insight into a Secretion Competent State of the Type Three Secretion System Needle Tip and its Interaction with Host Ligands
Source: PLoS Pathog. 2011 Aug 4;7(8):e1002163. doi: 10.1371/journal.ppat.1002163 (PMC3150277; doi:10.1371/journal.ppat.1002163)

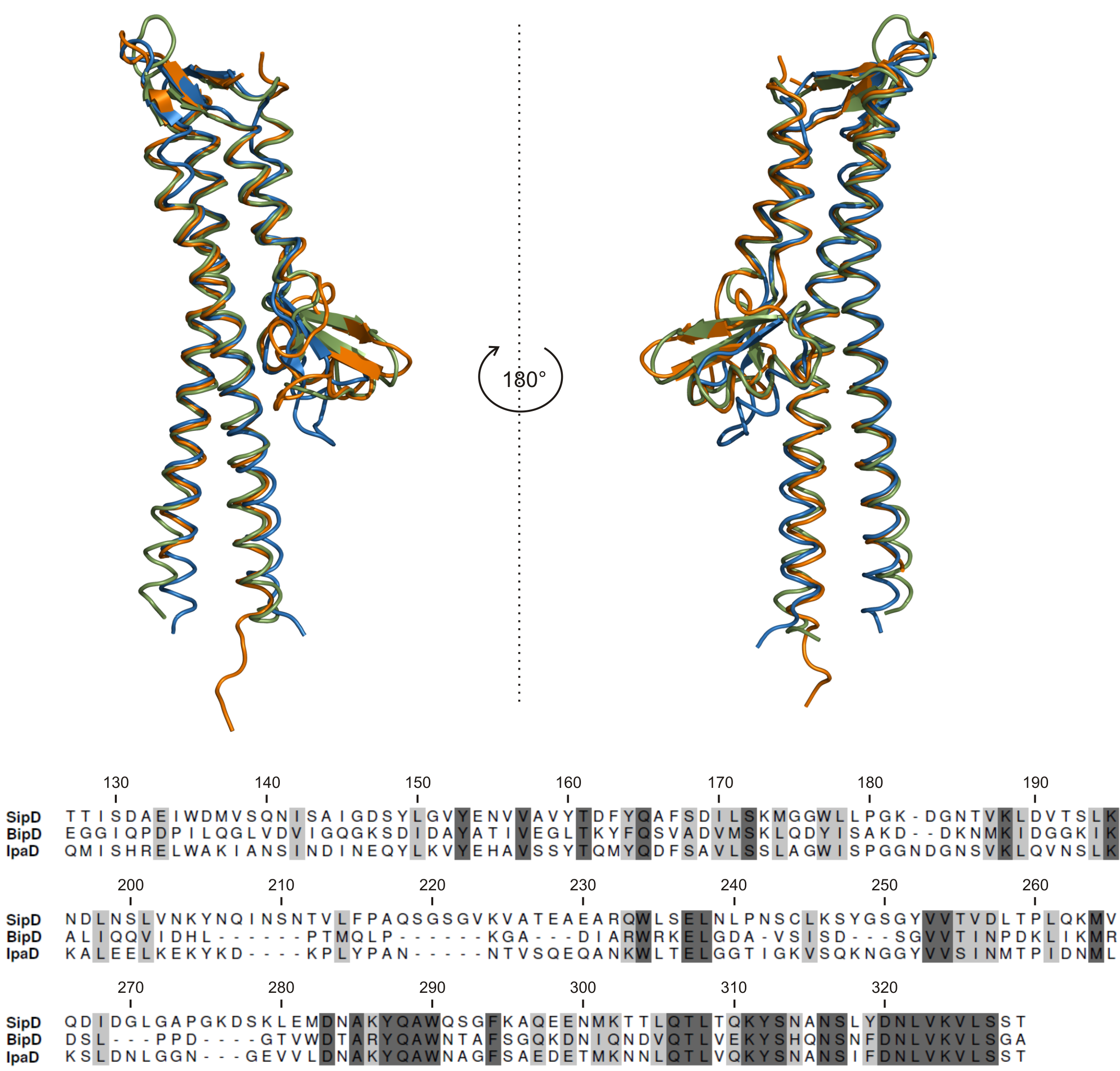

Supplement: Figure S1 — Structural conservation of T3SS needle tip proteins. Superposition of the domains 2 and 3 of the crystal structures of SipD from Salmonella (orange, this work), BipD from Burkholderia (blue, PDB code 2IZP), and IpaD from Shigella (green, PDB code 2J0O) and corresponding structure based protein sequence alignment (below). Identical and similar amino acids are highlighted in dark and light grey, respectively. Amino acids are numbered according to the SipD sequence. (TIF) [file ppat.1002163.s001.tif]

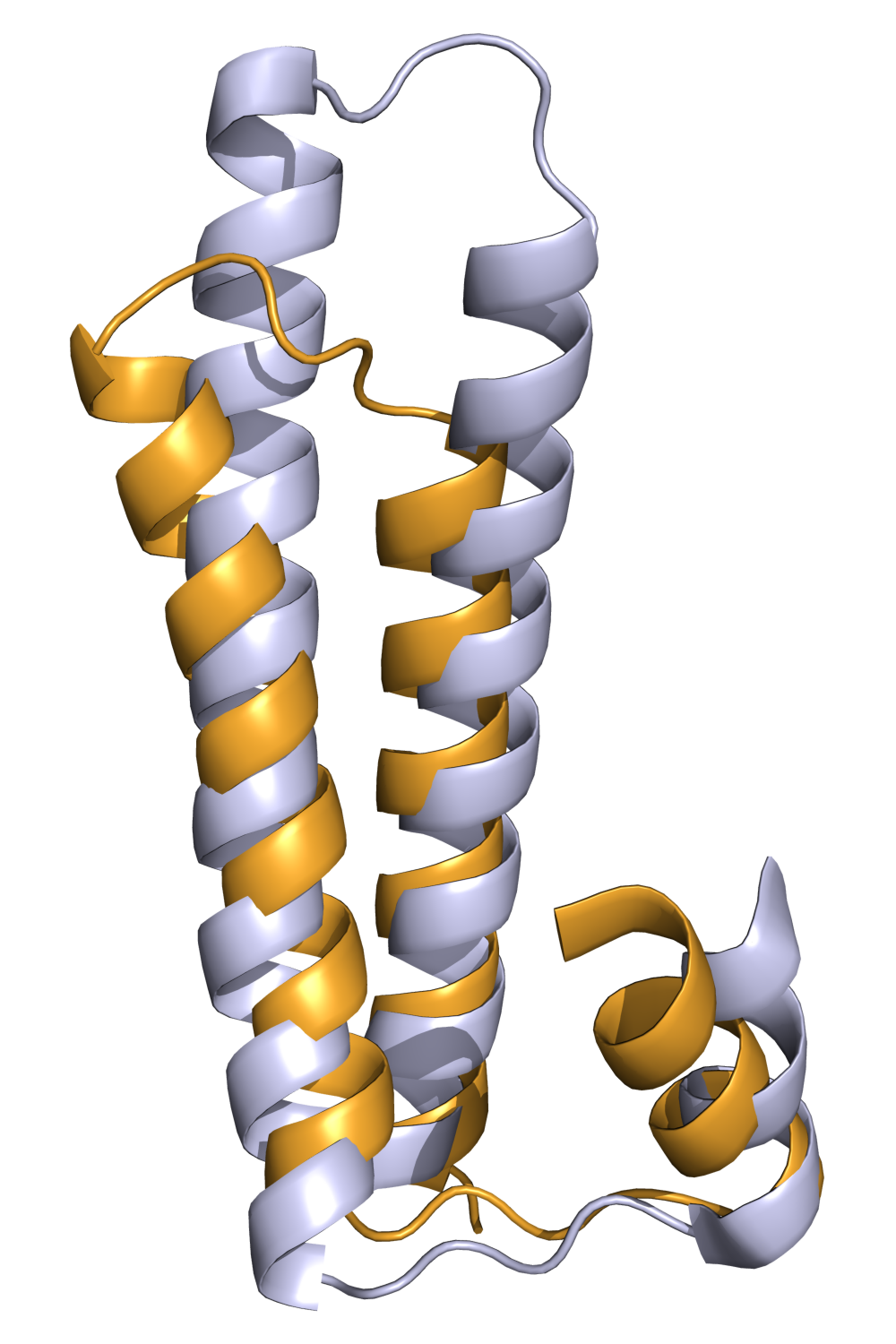

Supplement: Figure S2 — Structural similarity of the tip protein in domain 1. Superposition of the N-terminal domains of SipD (orange. this work) and IpaD (light blue, pdb code: 2J0O). (PNG) [file ppat.1002163.s002.png]

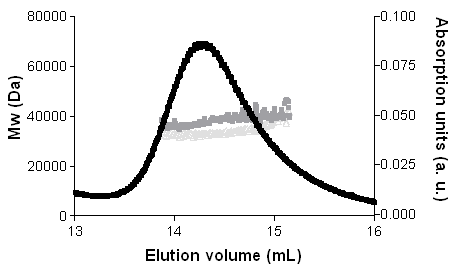

Supplement: Figure S3 — SipD is a monomer in solution. On-line static laser light scattering experiments of SipD eluted from a size exclusion column. The black line shows the protein absorption at 280nm (right axis) versus the eluted volume, indicating the presence of SipD. The light and dark grey lines refer to the left axis and reflect the measured molecular weight of SipD in solution for 2 independent experiments. (TIF) [file ppat.1002163.s003.tif]

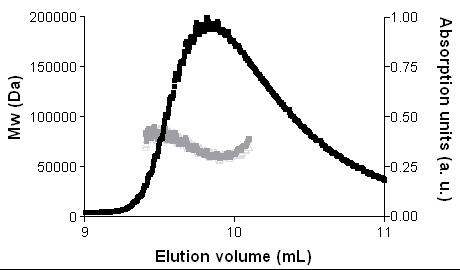

Supplement: Figure S4 — Deletion of the N-terminal domain 1 in SipD does not facilitate self-polymerization. On-line static laser light scattering experiments of SipDΔD1 eluted from a size exclusion column. The black line show the protein absorption at 280nm (right axis) versus the eluted volume indicating the presence of SipDΔD1. The light and dark grey lines refer to the left axis and reflect the measured molecular weight of SipDΔD1 in solution for 2 independent experiments. (TIF) [file ppat.1002163.s004.tif]

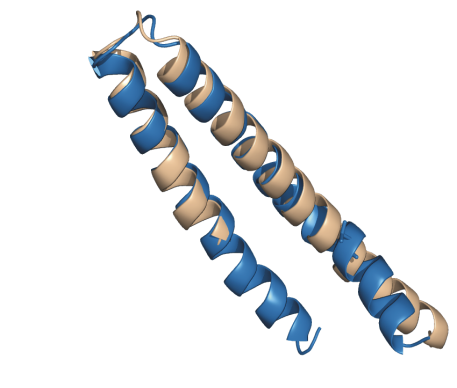

Supplement: Figure S5 — Conformational changes during interaction of SipD and PrgI. Superposition of PrgI as in the fusion protein (blue) with monomeric PrgI* (light brown) indicated structural differences in the C-terminal helix of the needle protein. (TIF) [file ppat.1002163.s005.tif]

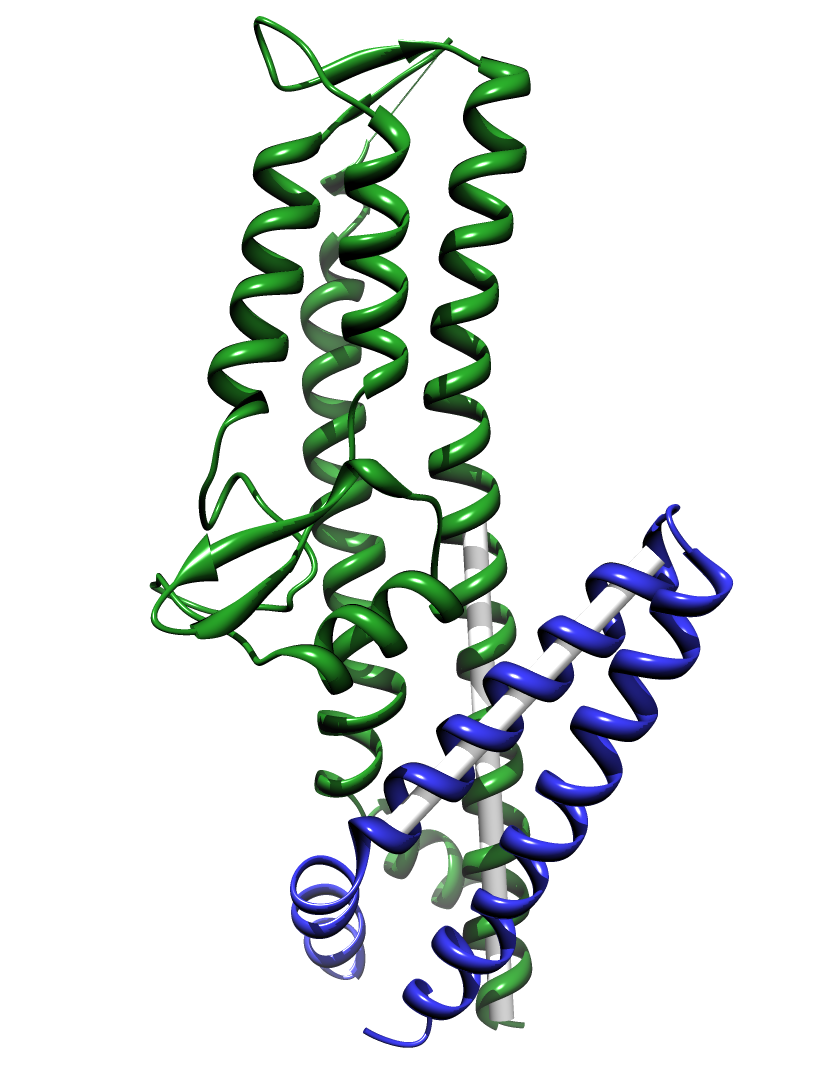

Supplement: Figure S6 — Relative orientation of SipD and PrgI in the fusion protein. SipD (green) and PrgI (blue) adopt a relative orientation of about 45°. Calculation is based on the relative orientation of the highlighted (grey cylinders) helices. (PNG) [file ppat.1002163.s006.png]

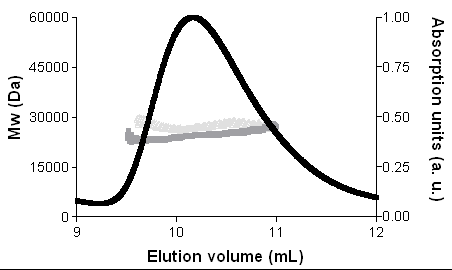

Supplement: Figure S7 — PrgI-SipDΔD1 fusion protein is a monomer in solution. On-line static laser light scattering experiments of PrgI-SipDΔD1 eluted from a size exclusion column. The black line show the protein absorption at 280nm (right axis) versus the eluted volume indicating the presence of PrgI-SipDΔD1. The light and dark grey lines refer to the left axis and reflect the measured molecular weight of the fusion protein in solution for two independent experiments. (TIF) [file ppat.1002163.s007.tif]

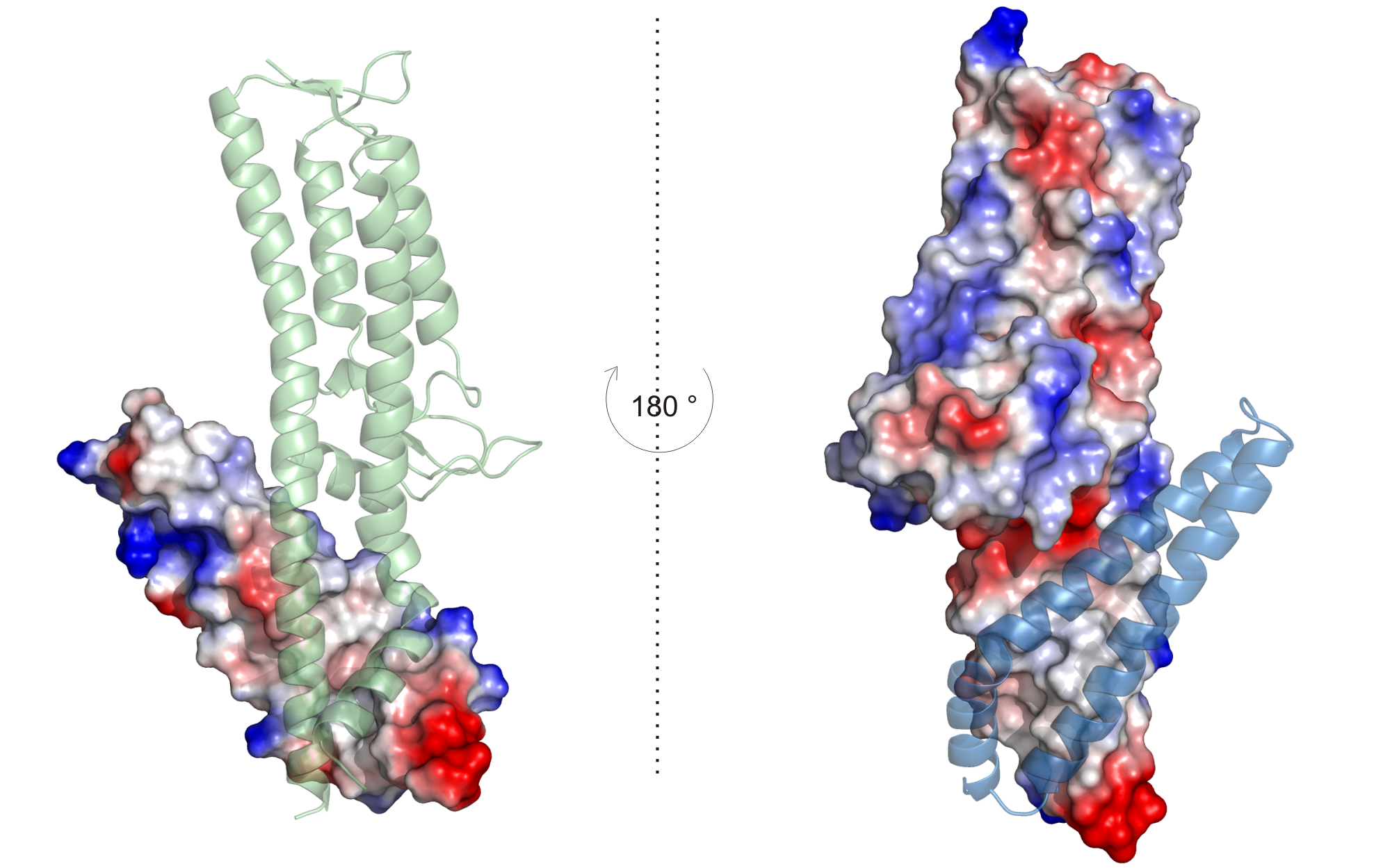

Supplement: Figure S8 — Hydrophobic surfaces stabilize the PrgI-SipDΔD1 fusion protein. Two perspectives of SipD (green cartoon on the left, surface representation on the right) and PrgI (surface representation on the left, blue cartoon on the right). Surfaces are coloured according to the electrostatic potential, blue: positive, red: negative). Uncharged surface patches at the interface between the two proteins indicate hydrophobic contacts. (TIF) [file ppat.1002163.s008.tif]

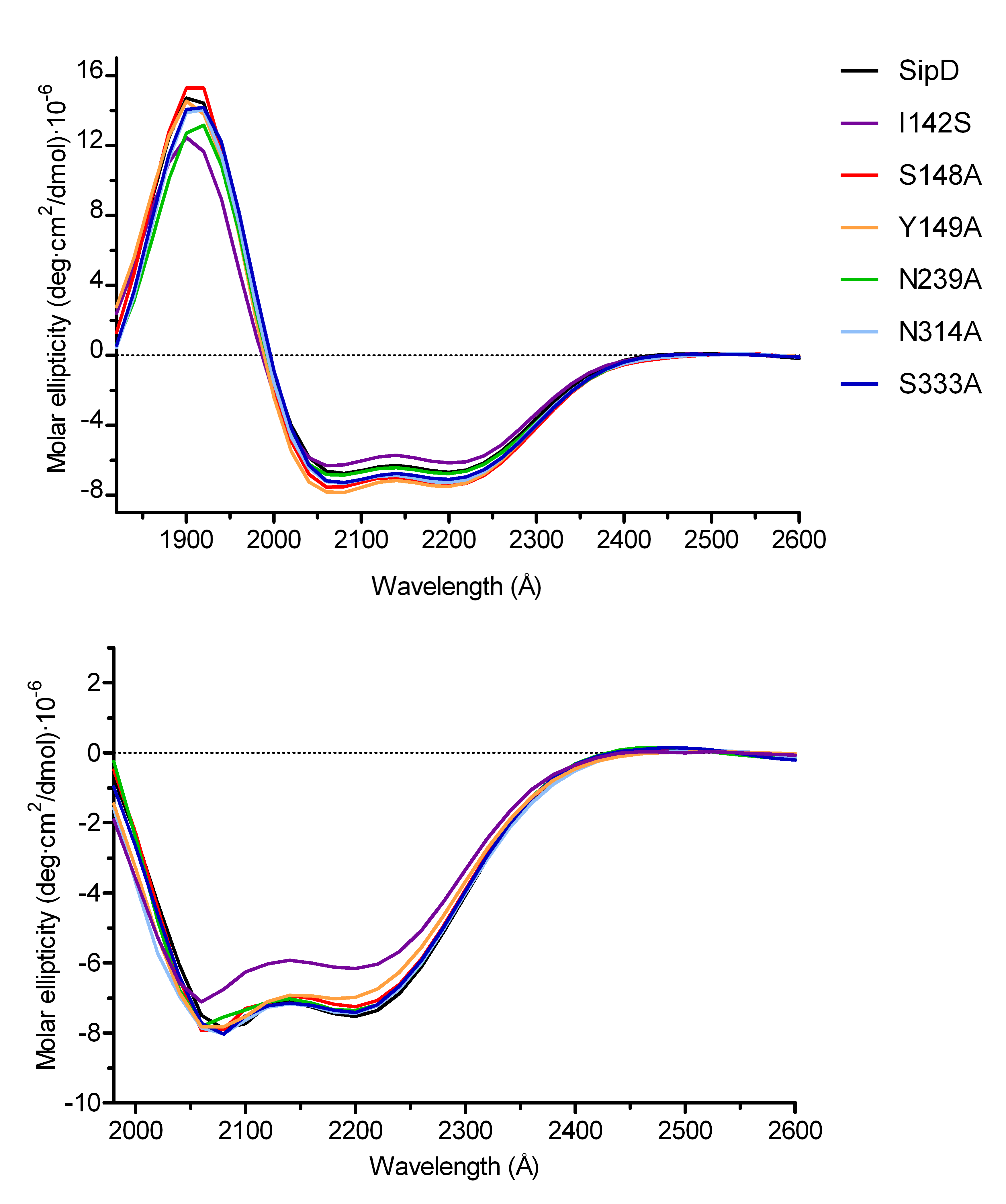

Supplement: Figure S9 — Circular Dichroism spectra obtained at 20°C and 37°C from purified SipD and SipD mutants. Except for I142S the spectra obtained from six mutants described in Figure 4 show similar secondary structure content at 20°C (upper panel) and at 37°C (lower panel). Mutant I142S shows reduced folding stability compared to wildtype, particularly at 37°C. Data at 37° (lower panel) were recorded to a lower limit of 1980 Å to avoid spectra distortion due to high photomultiplier voltage obtained with a temperature controlled cuvette with 1 mm optical path length. (TIF) [file ppat.1002163.s009.tif]

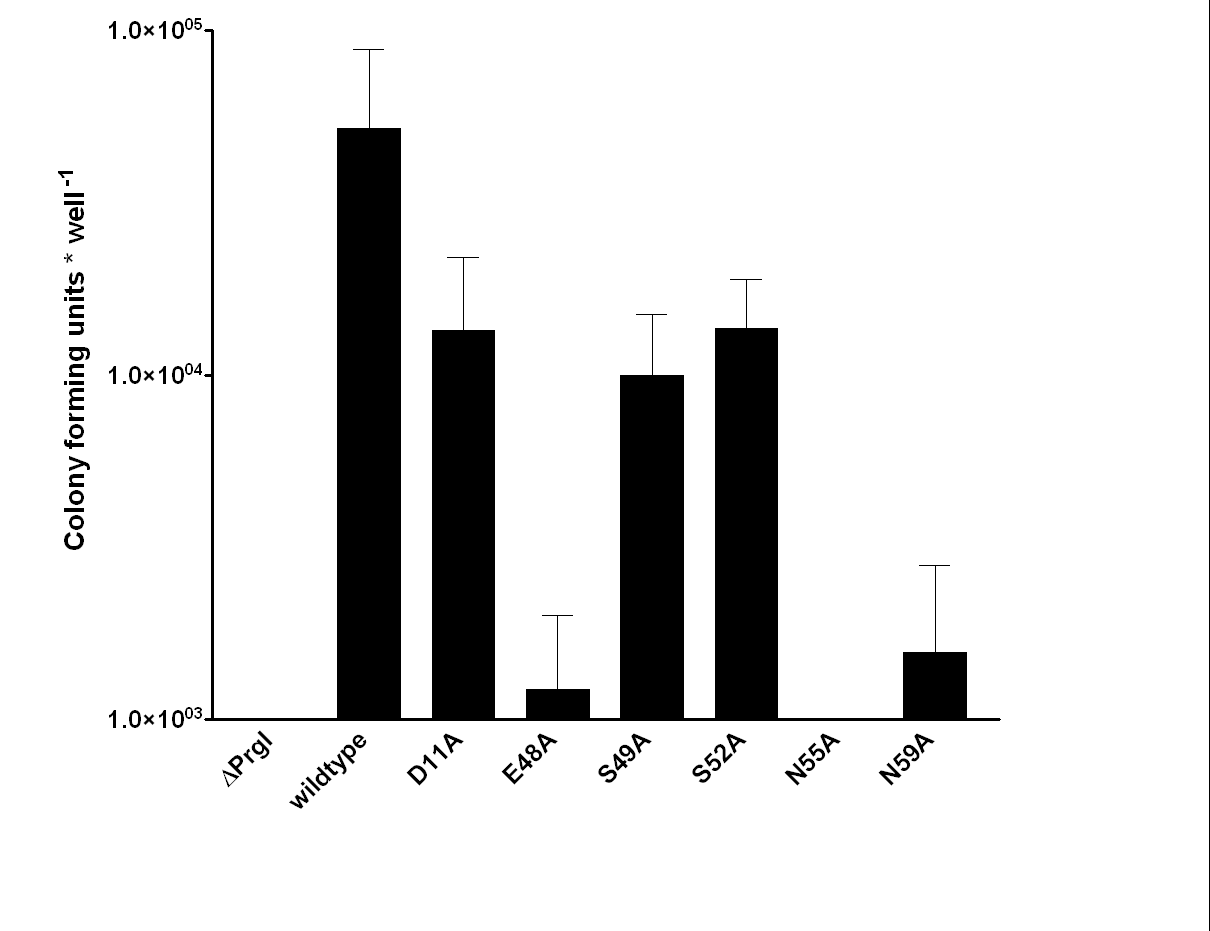

Supplement: Figure S10 — Host invasion assay of PrgI mutant complemented S. typhimurium knockout cells. (TIF) [file ppat.1002163.s010.tif]

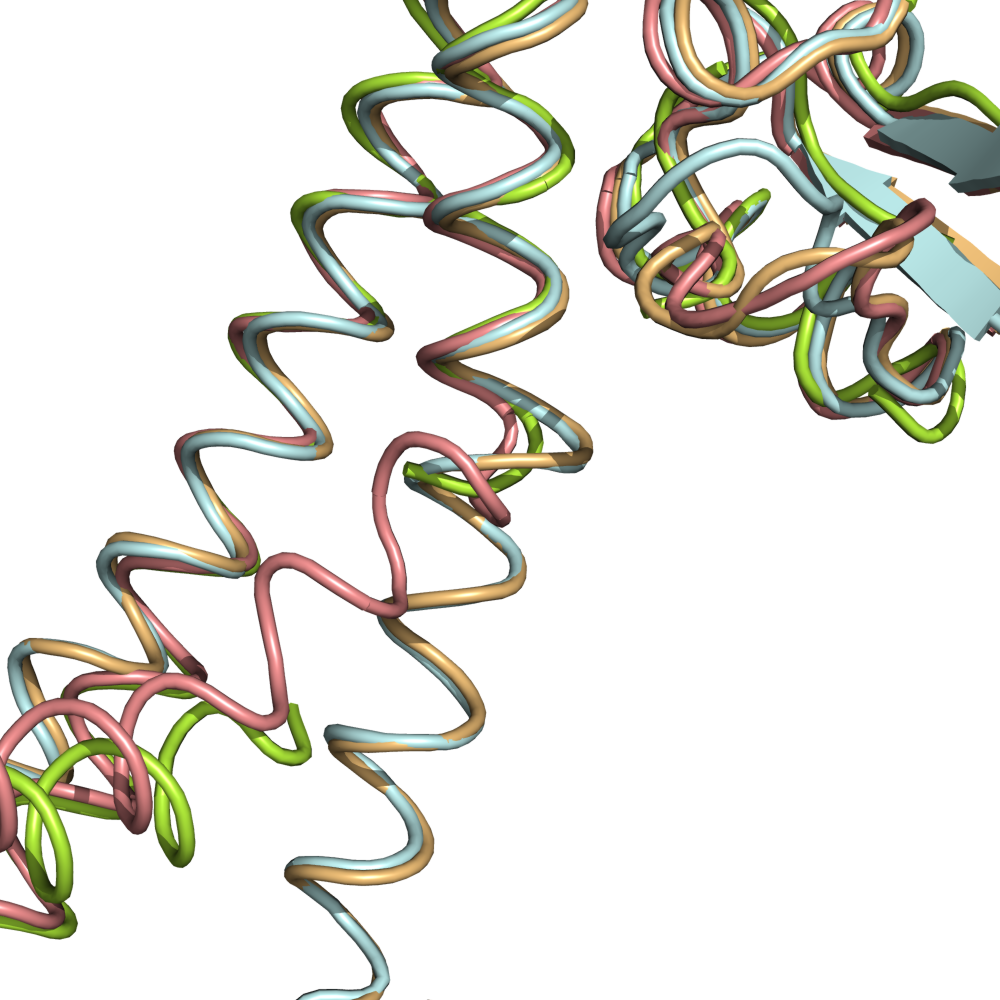

Supplement: Figure S11 — Superposition of the different copies found in the SipD crystal structure. Chain A (orange), chain B (light blue), chain C (red), and chain D (green) were superimposed and the region of the coiled-coil around Ser148 is highlighted. Chains A and B show a π-bulge, chain C and D are partially destabilized and kinked at the same position as SipD in the fusion protein. (PNG) [file ppat.1002163.s011.png]

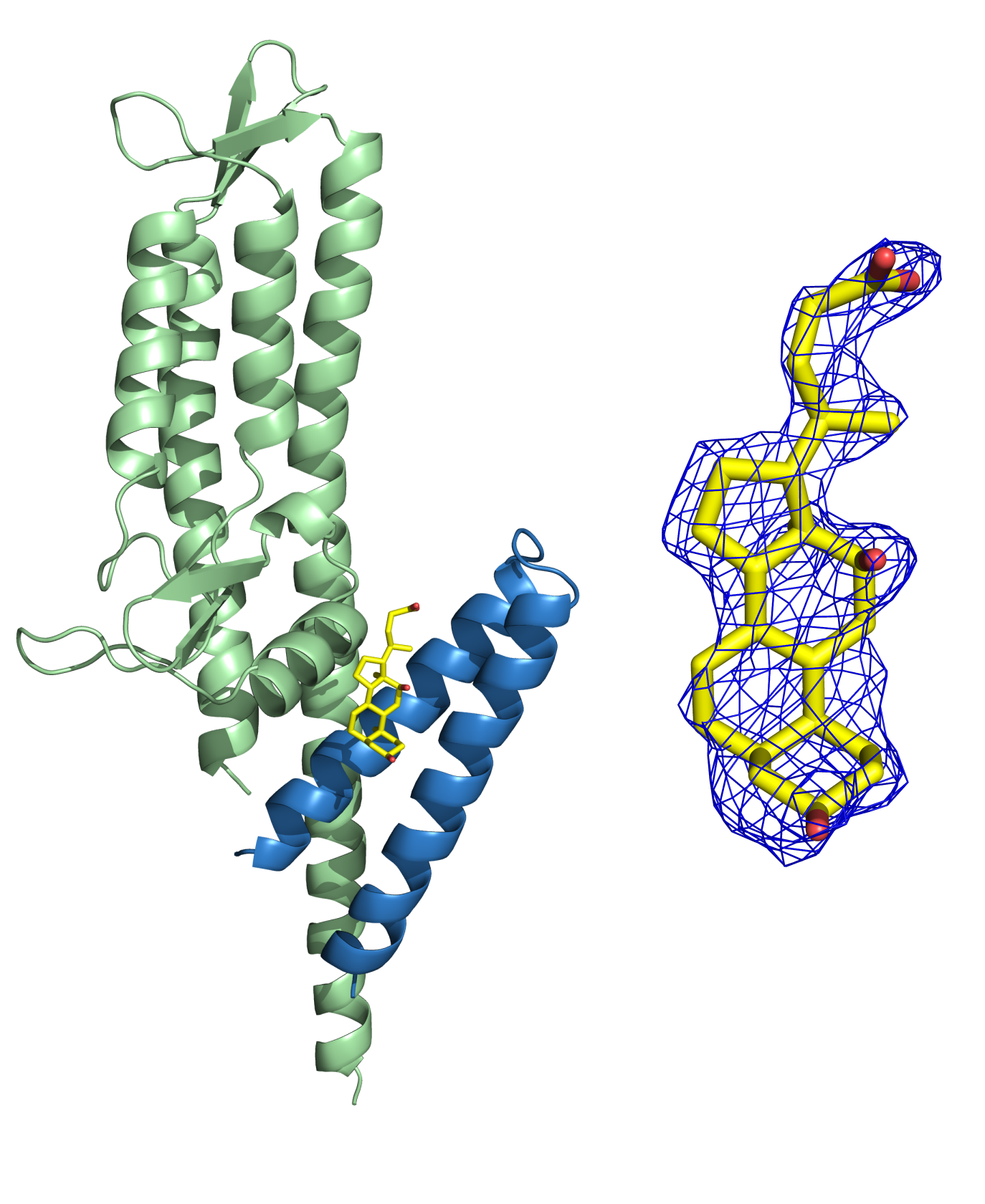

Supplement: Figure S12 — Co-crystal structure of PrgI-SipDΔD1 with deoxycholate. Left: Ribbon presentation of the PrgI-SipDΔD1 fusion protein (chain B, SipDΔD1: green, PrgI: blue) in complex with deoxycholate (yellow); Right: Bound deoxycholate in the same orientation as on the left shown with superimposed composite 2fo-fc density map (blue) contoured at 1 σ. (TIF) [file ppat.1002163.s012.tif]
